# Complete Genome Sequence of a Betanodavirus Isolated from Half-Smooth Tongue Sole (*Cynoglossus semilaevis*)

**DOI:** 10.1128/genomeA.01312-14

**Published:** 2014-12-18

**Authors:** Jin Li, Cheng Yin Shi, Jie Huang, Wei Guang Geng, Sheng Qiang Wang, Zi Dan Su

**Affiliations:** a Yellow Sea Fisheries Research Institute, Chinese Academy of Fishery Sciences (CAFS), Key Laboratory of Sustainable Development of Marine Fisheries, Ministry of Agriculture, Qingdao, China; b Shanghai Ocean University, Shanghai, China

## Abstract

Betanodavirus, commonly called nervous necrosis virus (NNV) of fish, has emerged as a major constraint on marine aquaculture worldwide. Here, we report the complete genome sequence of a betanodavirus (strain CsCN128) isolated from diseased half-smooth tongue sole (*Cynoglossus semilaevis*) in China. The genome sequence of strain CsCN128 shares ≥98.7% similarity with seven-band grouper nervous necrosis virus from Japan. Phylogenetic analysis indicates that strain CsCN128 belongs to the red-spotted grouper nervous necrosis virus (RGNNV) genotype of betanodavirus. The genome of the strain CsCN128 will facilitate further study on the molecular epidemiology and natural susceptible host range of betanodaviruses.

## GENOME ANNOUNCEMENT

Betanodavirus, commonly called nervous necrosis virus (NNV) of fish, is a major causative agent of disease in marine fish. It usually causes severe mortalities (up to 100%) of larvae and juveniles in aquaculture. Betanodaviruses are distributed worldwide and naturally affect >50 fish species (1, 2). However, half-smooth tongue sole (*Cynoglossus semilaevis*) has not been described as the host of betanodavirus up to now. In August 2012, an outbreak of disease caused by *C. semilaevis* broke out in a hatchery in northern China. Based on the clinical signs and virus detection, we confirmed that a betanodavirus (strain CsCN128) was the causative agent of the disease (3). It means that *C. semilaevis* is a new natural susceptible host of betanodavirus.

In order to determine the molecular characterization of strain CsCN128, we sequenced the complete genome of the virus. The naturally infected larvae (35 days posthatching) of half-smooth tongue sole were collected from a hatchery in China. Based on known betanodavirus nucleotide sequences, two pairs of reverse transcription-PCR (RT-PCR) primers were selected for amplification of the middle fragments of the strain CsCN128 genome (RNA1 and RNA2, respectively) (4, 5). Overlapping fragments covering the complete genome were subsequently amplified. The extreme 5′ and 3′ ends of the strain CsCN128 genome were amplified using the Rapid Amplification of cDNA Ends (RACE) (6, 7). Finally, combining the results from RT-PCR and RACE, the entire nucleotide sequences of strain CsCN128 were obtained.

The complete genome of strain CsCN128 consists of two single-stranded molecules of positive-sense RNA (RNA1 and RNA2). The RNA1 of CsCN128 is 3,103 nucleotides (nt), and the G+C content is 52.5%. It begins with a 5′ untranslated region (UTR) (nt 1 to 78), followed by an open reading frame (ORF) (nt 79 to 3027) and the 3′ UTR (nt 3028 to 3103). The RNA2 of CsCN128 is 1,433 nt, and the G+C content is 54.5%. It begins with a 5′ UTR (nt 1 to 26), followed by an ORF (nt 27 to 1043) and the 3′ UTR (nt 1044 to 1433). The subgenomic RNA3 of betanodavirus was also found in strain CsCN128. It is located downstream of RNA1 (nt 2753 to 2980).

The genomic sequence alignments showed that RNA1 and RNA2 of strain CsCN128 share 96.3% to ~99.1% homologies with those of the red-spotted grouper nervous necrosis virus (RGNNV) genotype isolates. Of these RGNNV genotype isolates, CsCN128 in China is most similar to seven-band grouper nervous necrosis virus in Japan (99.1% similarity for RNA1 and 98.7% similarity for RNA2) (8, 9). In contrast, CsCN128 shares only 79.1% to ~83.5% homologies across the entire genome with the genomes of other genotype isolates of betanodavirus. Phylogenetic tree analysis reveals that strain CsCN128 belongs to the RGNNV genotype of betanodavirus. These results will facilitate further study on the molecular epidemiology and natural susceptible host range of betanodavirus.

### Nucleotide sequence accession numbers.

The complete genome sequence of the RGNNV strain CsCN128 has been deposited in GenBank under the accession numbers KJ541747 and KJ541748.

## References

[1] OIE 2013 Chapter 2.3.11. Viral encephalopathy and retinopathy. World Organisation for Animal Health (OIE), Paris, France http://www.oie.int/fileadmin/Home/eng/Health_standards/aahm/current/2.3.11_VER.pdf.

[2] Munday BL Kwang J Moody N 2002 Betanodavirus infections of teleost fish: a review. J. Fish Dis. 25:127–142. 10.1046/j.1365-2761.2002.00350.x.

[3] Li J 2014 Research on viral nervous necrosis of half smooth tongue sole. Master of agriculture extension. Shanghai Ocean University, Shanghai, China (In Chinese with English abstract).

[4] Ransangan J Manin BO Abdullah A Roli Z Sharudin EF 2011 Betanodavirus infection in golden pompano, *Trachinotus blochii*, fingerlings cultured in deep-sea cage culture facility in Langkawi, Malaysia. Aquaculture 315:327–334. 10.1016/j.aquaculture.2011.02.040.

[5] Xu HD Feng J Guo ZX Ou YJ Wang JY 2010 Detection of red-spotted grouper nervous necrosis virus by loop-mediated isothermal amplification. J. Virol. Methods 163:123–128. 10.1016/j.jviromet.2009.09.009. 19755132

[6] Fu GK Wang JT Yang J Au-Young J Stuve LL 2004 Circular rapid amplification of cDNA ends for high-throughput extension cloning of partial genes. Genomics 84:205–210. 10.1016/j.ygeno.2004.01.011. 15203218

[7] Li Z Yu M Zhang H Wang HY Wang LF 2005 Improved rapid amplification of cDNA ends (RACE) for mapping both the 5′ and 3′ terminal sequences of paramyxovirus genomes. J. Virol. Methods 130:154–156. 10.1016/j.jviromet.2005.06.022. 16076500

[8] Takizawa N Adachi K Kobayashi N 2008 Establishment of reverse genetics system of betanodavirus for the efficient recovery of infectious particles. J. Virol. Methods 151:271–276. 10.1016/j.jviromet.2008.04.002. 18508134

[9] Iwamoto T Okinaka Y Mise K Mori K Arimoto M Okuno T Nakai T 2004 Identification of host-specificity determinants in betanodaviruses by using reassortants between striped jack nervous necrosis virus and sevenband grouper nervous necrosis virus. J. Virol. 78:1256–1262. 10.1128/JVI.78.3.1256-1262.2004. 14722280 PMC321384

